# Impact of United States refugee ban and discrimination on the mental health of hypertensive Arabic-speaking refugees

**DOI:** 10.3389/fpsyt.2023.1083353

**Published:** 2023-08-10

**Authors:** Behnan Albahsahli, Lana Bridi, Raghad Aljenabi, Dania Abu-Baker, Dahlia A. Kaki, Job G. Godino, Tala Al-Rousan

**Affiliations:** ^1^Herbert Wertheim School of Public Health and Human Longevity Science, University of California, San Diego, San Diego, CA, United States; ^2^School of Medicine, University of California, San Diego, San Diego, CA, United States; ^3^School of Social Work, San Diego State University, San Diego, CA, United States; ^4^School of Medicine, University of California, San Francisco, San Francisco, CA, United States; ^5^Laura Rodriguez Research Institute, Family Health Centers of San Diego, San Diego, CA, United States

**Keywords:** displacement and health, travel ban, Muslim ban, family separation, islamophobia, racism

## Abstract

**Background:**

Hypertension is a global leading cause of death which disproportionately affects refugees. This chronic disease increases the risk of heart disease, stroke, brain, and other end-organ disease, if left uncontrolled. The 2017 United States travel or “Muslim” ban prevented immigrants and refugees from seven Muslim-majority countries from entering the United States, including Syria and Iraq; two major contributors to the global refugee population. As of 2020, the United States has admitted more than 133,000 and 22,000 Iraqi and Syrian refugees, respectively. Studies on the health effects of this policy on refugees are lacking. This study qualitatively explores the impact of the refugee ban on United States resettled Syrian and Iraqi refugees with hypertension.

**Methods:**

Participants were recruited through a federally qualified health center system that is the largest healthcare provider for refugees in San Diego, CA. All participants were Arabic-speaking refugees diagnosed with hypertension from Syria and Iraq. In-depth interviews took place between April 2021 and April 2022. Inductive thematic analysis was used to analyze data from semi-structured interviews.

**Results:**

Participants (*N* = 109) include 53 women and 56 men (23 Syrian, 86 Iraqi). The average age was 61.3 years (SD: 9.7) and stay in the United States was 9.5 years (SD 5.92). Four themes emerged linking the travel ban’s impact on health, in line with the society to cells framework: (1) family factors: the refugee ban resulted in family separation; (2) physiological factors: the refugee ban worsened participants’ mental health, exacerbating hypertension and perceived health outcomes; (3) community factors: perpetuation of Islamophobia, xenophobia, and perceived discrimination were structural barriers with links to poorer health; and (4) individual factors: trickle down consequences led to worsened participant self-image and self-perception within their host community.

**Discussion:**

The refugee ban negatively impacted the mental and physical health of United States resettled Arabic-speaking refugees through perceived discrimination, stress, and poor social integration. It continues to have long-lasting effects years after the ban was instated. Centering family reunification within the United States Refugee Admissions Program and tailoring interventions through the healthcare and public health systems are warranted to reduce hypertension disparities in this growing and overlooked population.

## 1. Introduction

In 2017, former President Trump signed Executive Order 13769, called “Protecting the Nation from Foreign Terrorist Entry into the United States.” This travel ban prevented immigrants and refugees from 7 Muslim-majority countries from entering the United States including those from Iran, Iraq, Libya, Somalia, Sudan, Syria, and Yemen (1). This travel ban is often referred to as the “Muslim ban” because it exclusively targeted those from Muslim-majority countries burdened with war and political conflict and is also interchangeably referred to as the “refugee ban.” In this manuscript, we will use the term refugee ban to refer to this policy. We will use the term “refugee ban” instead of “travel ban” to avoid confusion with other travel bans, including, most recently, the travel restrictions associated with the COVID-19 pandemic. The executive order, which lasted for 2 years, suspended the United States Refugee Admissions Program (USRAP) for 120 days to allow the administration to review the applications and ensure incoming refugees do not pose a “threat to the security and welfare of the United States” (2). Consequently, the number of refugees admitted to the United States in 2017 saw a 66% drop from the number of refugee arrivals in 2016 – the lowest number of refugees admitted to the United States since 1977 (3). The number of refugee admissions to the United States remains significantly less than the number of refugees admitted prior to 2016 (4). As of 2020, more than 133,000 Iraqi and 22,000 Syrian refugees have been admitted to the United States (5–7).

Due to the racialization of Islam and the rise of Islamophobia globally, the majority of individuals from the Middle East and South Asia are stereotyped as Muslim and can be subjected to discrimination, injustice, and hate (8). The refugee ban was criticized by policy experts and health advocates for being Islamophobic and discriminatory (2, 9). In addition to unjustly restricting migration for forcibly displaced Middle Eastern and North African (MENA) refugees, learning from our study found the refugee ban to be impacting the physical and mental health of refugees who are targeted by the ban. Abundant literature has shown that racial discrimination negatively impacts both mental and physical health, and is intricately tied to worse health behaviors, poor adherence to medical regimens, and limited utilization of healthcare services (10). Precisely, perceived discrimination is associated with a higher prevalence of hypertension and racism can exacerbate hypertension due to the restriction of available resources and the creation of a social sense of inferiority (11, 12). One systematic review conducted in 2018 found Islamophobia to be consistently associated with poor mental health such as greater depressive symptoms and anxiety about discrimination in Muslim and in Middle Eastern and South Asian populations. It also found Islamophobia to be associated with poor health behaviors like a lack of physical activity and poor nutrition. Additionally, it found that Islamophobia was negatively associated with care-seeking behaviors and limited access to healthcare services (8). As for refugees, experiences of perceived racial discrimination have been found to be associated with greater levels of depression among refugees reporting this exposure than among those that have not (13). Additionally, other studies have found experiences of perceived discrimination to not only be associated with depressive symptoms, but also symptoms of PTSD (14).

Refugees are burdened with health disparities and barriers to healthcare access, and have a particularly high prevalence of hypertension (15, 16). Prevalence rates of hypertension among refugees vary widely due to factors like location and size of the study, highlighting the need for large scale prevalence studies. A literature review investigating the prevalence of HTN among refugees in the Middle East found Iraqi refugees had a prevalence of hypertension between 19.6 and 22% (16). The article also found the prevalence of HTN among Syrian refugees to vary depending on the country of resettlement, where rates were higher than 50% in Lebanon, 32% in Turkey, and 9.7% in Jordan (16). Hypertension is a global leading cause of death, killing more than 7.5 million individuals around the world each year (17–19). When poorly controlled, HTN can result in detrimental outcomes like stroke or heart disease (20). Refugees are particularly challenged by the poor management of this chronic disease due to challenges with accessing healthcare services and a lack of knowledge surrounding the care for this condition (16).

Some studies have described the association between refugees’ mental ill health and the disruption of social networks (21, 22). Saadi et al. have found that after the issuance of the Muslim or refugee ban executive order, missed primary care appointments and ED visits increased among people from Muslim-majority countries living in Minneapolis-St. Paul (23). However, there is no formal research documenting the impact of the refugee ban on hypertensive refugees or their management of chronic conditions. With the loss of social networks due to forced displacement, it is critical to understand how the refugee ban impacted the health and well-being of Arabic-speaking refugees with hypertension. Although the literature on the impact of discrimination on health exists, there is a research gap documenting the impact of discrimination on the health of refugees, particularly because of the refugee ban. Through in-depth interviews and analysis, this paper investigates the effects of the 2017 refugee ban on Arabic-speaking Syrian and Iraqi refugees with hypertension in San Diego, CA, United States.

**Figure 1:**
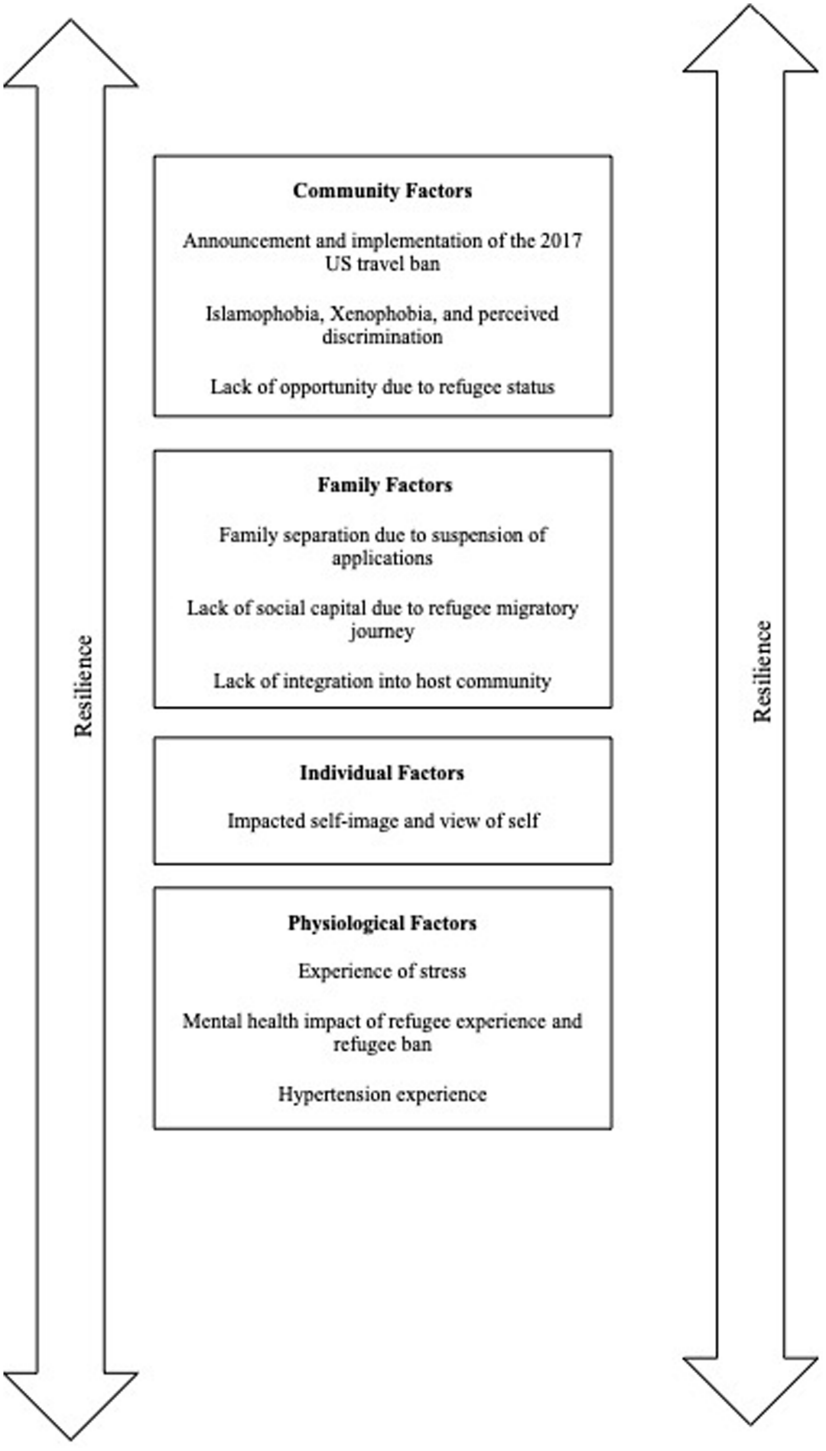
Themes organized in line with the society-to-cells framework.

This paper utilizes the society to cells (STC) resilience framework (24) as the theoretical underpinnings in the thematic analysis (24). This model theorizes the various tenets that may influence a particular person’s capacity and ability for resilience when faced with various challenges. Resistance encompasses: the ability of a person to resist poorer health challenges (like hypertension), the ability to recover from challenges, and the ability to return to a better state of health prior to the onset of the challenge (25). The tenets that hold the capacity to both facilitate and reduce resilience include: societal factors, community factors (like institutions and social capital), familial factors (like connectedness and sense of safety/security), individual factors (race, view of self, view of the world) physiological factors (like neurochemical activity and other physiological responses) and cellular factors (mechanisms at the cellular level). It is important to note that these tenets hold the ability to interact with one another. Here, we theorize the impact of these various tenets on the resilience of refugees with regard to addressing the challenge of managing their hypertension diagnoses (Figure 1).

## 2. Materials and methods

### 2.1. Setting and subjects

Participants were recruited between April 2021 and April 2022 through Family Health Centers of San Diego (FHCSD) – a federally qualified health center system and a large provider for refugees in San Diego — and the Majdal Community Center (MCC) – an ethnic-based community organization and study partner. This study is a sub-study from a larger, parent study and intervention investigating the feasibility of self-managing hypertension for refugee groups. Participants were selected randomly from a patient list provided through FHCSD and contacted *via* phone call. Criteria for inclusion in the study were: (1) having refugee status at any point in time, (2) having a hypertension diagnosis verified through the electronic health records at FHCSD, (3) being from Iraq or Syria, and (4) living in San Diego. California is home to the largest Arab American Diaspora and San Diego has been a top destination for refugees resettled in California (26–30). This study only included refugees from Iraq and Syria because of this study’s focus on refugees from the Middle East, as well as Syrian and Iraqi refugees making up the largest demographic of Middle Eastern refugees in San Diego (31). Exclusion criteria for the study were: (1) being under the age of 21 and (2) not being able to provide informed consent. Interviews were scheduled with enrolled participants at the MCC or on Zoom (based on local COVID-19 guidelines) and participants were compensated for their participation at the end of the study. There were 756 calls made to random patients on the FHCSD patient list. Of the 756 individuals contacted, 535 declined to participate, 47 did not meet the inclusion criteria, and 41 belonged to an overrepresented study demographic (Iraqi) relative to the local population in San Diego and were not included in the study. 133 individuals agreed to participate, 20 withdrew, and 4 were removed by study staff. One hundred and nine participants were included in the study to meet the requirements for the parent, intervention study.

### 2.2. Data collection

Demographic information including age, gender, date of resettlement in the United States, education, marital status, and income were collected. Semi-structured interviews were conducted in Arabic or English depending on the participant’s preference. Interviews were recorded with patient consent then transcribed and translated into English by a certified translator. The certified translator would reproduce the entire conversation with the participant in writing without omitting any portion of the conversation. Interviews were conducted by trained research assistants fluent in Arabic and English and share cultural background with the participants (BA, LB, DA). The interview guide was developed by experts after having conducted a literature review to study the health and well-being of hypertensive refugees (Appendix A). Considering this study is a sub-study from an investigation into the knowledge, attitudes, and behaviors of refugees surrounding their hypertension management, there was one, primary question investigating the impact of the refugee ban on health. The subsequent questions following the structured question shared in Appendix A were unstructured and based on the participant’s response.

### 2.3. Data analysis

All 109 participants were included in the quantification of demographic variables. Inductive thematic analysis was used to analyze qualitative data. Forty-two participant interview transcripts were included in the qualitative analysis. Interviews were coded independently by three team members using ATLAS.ti. This software analyzes transcript document files. All of the coders have past experience working with published qualitative research and have extensive experience working in public health research with this population. Transcripts were first coded to deduce all the themes present in the data, then re-coded to further code the details pertaining to the refugee ban. Data analysis was conducted using Crabtree and Miller’s 5-step interview method to recognize recurring themes (32). This process repeated until the saturation of themes was attained. Results were reported utilizing the Standards for Reporting Qualitative Research (33).

### 2.4. Reliability and validity

We employed the criteria of Cohen and Crabtree (34) to enhance the rigor of our qualitative methods (34). To address reliability and reduce the potential for error and differences when analyzing the transcripts, the team members met regularly to establish a codebook. This process allowed for the coders to peer-review the findings in the qualitative analysis. To address the validity of our research, we emphasized the importance of being from the community throughout the research process. Therefore, the researchers conducting the interviews and the data analysis were Arabic-speaking Syrian and Iraqi with contextual knowledge on the participant population and MENA region.

### 2.5. Ethics approval

This study was approved by the Institutional Review Board at the University of California, San Diego. IRB (# 200063).

## 3. Results

### 3.1. Demographics

Data on participants’ demographic information is provided in Table 1. A total of 109 participants were included in this study, 23 (21.1%) were from Syria and 86 (78.9%) were from Iraq. The average age of participants was 61.3 years old (SD 9.7). The average length of resettlement in the United States was 9.5 years (SD 5.9). Of the participants, 56 (51.4%) were men and 53 (48.6%) were women. There were varying levels of education among the participants in this sample, with 43 (39.4%) attaining less than high school and 26 (23.9%) attaining a high school diploma. 94 (86.2%) of the participants in this sample were unemployed and 66 (60.6%) of the participants had an annual household income of less than $15,000. 91 (83.5%) of the participants in this sample were married. The thoughts and feelings of participants in the data supporting the qualitative findings have been quantified in Table 2.

**Table 1:** Demographic information for Syrian and Iraqi hypertensive refugees in study sample.

Demographics	Frequency	Percentage
**Sex (*n* = 109)**		
Male	56	51.4%
Female	53	48.6%
**Age**		
35–44	5	4.6%
45–54	19	17.4%
55–64	45	41.3%
65+	40	36.7%
**Marital status**		
Married	91	83.5%
Divorced	3	2.8%
Widowed	14	12.8%
Never Married	1	0.9%
**Country of origin**		
Syria	23	21.1%
Iraq	86	78.9%
**Employment**		
Employed	15	13.8%
Not Employed	94	86.2%
**Highest level of education attained**		
Less than high school	43	39.4%
High school	26	23.9%
Vocational certificate	16	14.7%
Bachelor’s degree or higher	24	22.0%
**Annual household income**		
Less than $15,000	66	60.6%
$15,001–$25,000	32	29.4%
$25,001 and higher	11	10.1%
**Number of hypertension medications**		
1	57	52.3%
2	26	23.9%
3 or more	17	15.6%
Data not available	9	8.3%

**Table 2:** Count of participants thoughts and feelings in qualitative results.

Participants thoughts and feelings	Count
Family members’ travel applications were not processed	14
Worse mental health due to refugee ban	15
Worry about family members barred from entry into the US	7
Worse health and hypertension due to refugee ban	8
Thinking about reunification with family members	7
The refugee ban perpetuated Islamophobia, Xenophobia, and discrimination	8
Experiences of racist encounters in the US	4
Policy is wrong and perpetuated stereotypes	7
Negative self-perception and understanding of themselves in host-country	3
Cynical outlook towards integration and experience of stress	4
Refugee status is obstructing integration and contributions, and not enough resources for refugees	6

### 3.2. Family factors: the refugee ban resulted in family separation

The majority of participants shared that the refugee ban resulted in them being separated from their families. This separation was due to the suspension of the travel applications that had been processed for years prior to the installment of this policy. This tenet of the STC framework negatively impacted the resilience potential of participants to manage their hypertension diagnoses. Participants shared detailed accounts of events following the implementation of the refugee ban:

“[The refugee ban] is the thing that has affected my health the most. My daughter who is a refugee in the Netherlands tried coming to the United States when I applied for her. The moment my daughter entered the United States embassy in the Netherlands they sent her back and refused her application.” Muslim Syrian Male Age 55

“When it happened, they did not let my son enter the United States my health was worse. And these policies stopped his paperwork.” Iraqi Female Age 56

Participants are working to renew the applications for their family members to reunite with them in the United States. However, these plans were intertwined with worries due to the potential failure of the application process, and worry for family members that they left behind:

“[The refugee ban] affected us a lot since I was waiting for my children in Jordan to come here. [The refugee ban] depressed us so much. However, I am looking forward to the Biden administration’s immigration policy, hopefully, they can come here.” Iraqi Male Age 67

“It affected me a lot. My eldest son was supposed to come here but the ban prevented him. I worry about him every day. [The refugee ban has definitely affected the mental health of refugees].” Non-Muslim Iraqi Female Age 61

### 3.3. Physiological factors: the refugee ban worsened participants’ mental health

Participants overwhelmingly reported having worse mental health as a result of the refugee ban, and their separation from their family members. The detrimental impact on mental health, or this physiological factor, also holds the capacity to negatively impact the resilience ability in the face of hypertension management. Participants shared feelings of distress and immense sorrow because of this policy.

“My wife and family were devastated. We tried again this year, and we are hoping it works so we can reunite with her.” Muslim Syrian Male Age 55

Several participants attributed their poorer mental states to the refugee ban and entertained family reunification as a remedy to their poor mental and physical states. Participants expressed hope regarding rejoining their family members.

“I got really depressed because my brothers did their interviews to come to the United States but once the Trump administration took office their travel was put on hold. I wish they could come; I would be so happy.” Muslim Syrian Male Age 55

Participants shared feelings of depression and anxiety due to refugee ban. Some reported seeking psychiatric help to overcome the mental health effects of the refugee ban:

“[The refugee ban] affected my mental health a lot. I am seeing a psychiatrist for my insomnia, nightmares, and forgetfulness. All because of what I went through.” Muslim Syrian Male Age 58

“[The refugee ban] affected me a lot, especially being away from so many relatives. Whenever I think about it, I get triggered.” Iraqi Female Age 61

### 3.4. Physiological factors: the ban and its associated stress exacerbated hypertension and perceived health outcomes

Participants also namely discussed the detrimental effects of the refugee ban on their on hypertension and chronic disease management as well as their perceived health outcomes. Participants discussed their overall health and well-being becoming worse due to the unfolding of these political events. Here, participants noted direct links between the challenge of their ability to maintain their health and the factors reducing their ability to do so.

“[The refugee ban] affected me so much, at that time my daughter was supposed to travel to the United States and this policy prevented her from visiting us. This drastically made me sicker, and I started visiting the hospital more often. I was dizzy all the time and had nervous breakdowns.” Muslim Syrian Female Age 48

“[The refugee ban has affected my health and well-being] due to anxiety and worry about bringing my daughter here. [This anxiety affected my hypertension].” Muslim Iraqi Female Age 58

Participants discussed the worsening of their hypertension due to stress and their worsening mental health resulting from the implementation of the refugee ban and the suspension of their family members’ travel applications to the United States:

“Personally, [I was impacted by the refugee ban] because many members of my family are waiting for their applications to be processed. My brothers and their families should have been here since 2014 but their applications got suspended. So, overall, my health deteriorated, and my hypertension became worse.” Muslim Iraqi Female Age 51

“Personally, I want my son to be reunited with me. Family reunification is the real medication for high blood pressure, not pills. He is my son, what is the reason for separating my son from me?” [Participant 20]

### 3.5. Community factors: islamophobia, xenophobia, racism, and perceived discrimination were seen as structural barriers with links to poorer health

Participants noted additional consequences resulting from the implementation of the refugee ban, including the perpetuation of Islamophobia, xenophobia, and discrimination. They indirectly described the implementation of this structural change as a display of institutional racism. These community factors, therefore, held the capacity to impede resilience to their hypertension management.

“[I absolutely felt that the refugee ban was unjust], after seeing all the horrors, this ban was very arbitrary and unnecessary.” Iraqi Female Age 50

“I really do not know why they implemented this racist law. It affected my mental health so much.” Muslim Syrian Female Age 61

Participants described various instances where they experienced racist encounters in the US, and attributed these racist incidents to the implementation of the refugee ban:

“[The refugee ban has affected us], especially the Islamophobia in the West. For example, when we were being interviewed for refuge in the United States, they were always cautious about who to admit and allow to come to the United States.” Muslim Syrian Female Age 39

“[Sometimes I feel unwanted when I realize the refugee ban is targeted against Syrians]. I have encountered racist people in the United States that look down upon us. They act like we are going to steal their resources and opportunities.” Muslim Syrian Male Age 47

The observance of hijab was often the marker of such racism, and some participants explicitly attributed the occurrence of these xenophobic incidents to their observance of the hijab -or the head covering observed by Muslim women. Participants shared how their family members that wear the hijab experienced bullying, which consequently impacted their health:

“[Refugees have the highest rates of hypertension because of] facing racism and discrimination in a foreign country. I encountered racist situations from my neighbors. And these racist incidents happened with both Arab Americans and non-Arab Americans. They bullied us for being refugees and my daughters were bullied at school which affected my mental health a lot. They bullied my daughters for wearing hijab.” Muslim Syrian Male Age 49

While only mentioned a few times, it is important to note that these Islamophobic encounters served as a structural barrier for these refugees to practice their faith publicly and observe their constitutionally protected first amendment rights:

“[I was always worried because] my youngest daughter was mistreated at school by her classmates because she was wearing a hijab. She was bullied at school for wearing the hijab so she took it off.” Muslim Syrian Female Age 39

Participants described this policy as wrong, and that it perpetuated stereotypes. Participants also felt it necessary to clear their name from the stereotypes perpetuated by this policy:

“[The refugee ban] bothered me, because of the stereotypes the ban perpetuated and advertised about us. We really come from a great nation and culture and not as primitive as the west tries to show.” Muslim Syrian Male Age 41

“[The refugee ban affected my mental health so much]. People are coming, being productive, and working. It is not like they want to come to the United States for vacation. Muslim Syrian Female Age 61

### 3.6. Individual factors: the refugee ban’s trickle-down consequences worsened refugees’ self-image, self-perception, and livelihood

Participants discussed that the refugee ban elicited a negative self-perception and a negative understanding of how other individuals in their host country perceived them. Participants discussed that the implementation of the refugee ban resulted in them feeling unwanted in the host country and resulted in a loss of self-worth. These variables are factors within the individual that hold the capacity to negatively impact the resilience to dealing with hypertension as a challenge.

“The word “refugee” itself bothers me and makes me feel inferior especially after leaving my country. And this feeling has a negative impact on my mental health and self-perception.” Muslim Syrian Male Age 55

“The idea of being a refugee makes me feel useless. I love being productive and it is hard being productive as a refugee in a foreign country after losing all your success and hard work in your country of origin. I would describe the feeling as an adult who is running around with a child’s mentality who is not being supported. I genuinely feel I aged so much in the United States I worked as an Uber driver even though I know I have so much potential to do more.” Muslim Syrian Male Age 54

Participants also described a feeling of powerlessness surrounding their ability to influence any political change:

“This is a political decision that was set forth by a country and I have no control over it.” Muslim Syrian Male Age 36

Refugee participants also described a cynical outlook toward their integration into the United States as refugees because of the refugee ban. Participants described feelings of worry and stress regarding their legal status, specifically around fear of deportation and inability to work:

“I feel like at any point I will be deported. Thus, it has increased my stress. When I am stressed, my blood pressure goes up and I go to the emergency room.” Iraqi Female Age 57

“I consistently experience mental fatigue, exhaustion, and anxiety. For example, right now I am too anxious since I am applying for United States citizenship and my English skills are weak. Thus, I am afraid it will be hard to get citizenship and assimilate.” Muslim Syrian Male Age 36

Participants shared that they feel refugees’ integration is actively being obstructed by the United States government. These feelings were heightened due to the refugee ban:

“You feel like this government is fighting you so hard… All of this has caused us anxiety and sadness.” Muslim Syrian Male Age 45

Participants shared the feeling that they are treated differently from United States citizens, and they described their refugee status as a barrier to their integration and their ability to contribute to society. They also described an inadequate number of resources provided to refugees to enhance their successful integration into the United States. These feelings of unwantedness and lack of freedom were exacerbated by the refugee ban:

“I can’t afford a bigger house and I am not paid enough per hour like United States citizens, above all that I am not equipped enough to navigate my way in the American society.” Muslim Syrian Male Age 45

“When we came here it was only for the first 1-3 months that we were taken care of and helped out. After that, we were all on our own and reality hit us hard. It is harder than we expected to live here and try to make it.” Muslim Iraqi Female Age 36

[My refugee experience] has caused me more stress, and I have no legal papers preventing me from having freedom of movement, traveling, or working.” Iraqi Female Age 57

## 4. Discussion

To our knowledge, this is the first study investigating the impact of the refugee ban on Syrian and Iraqi refugees resettled in the United States with a diagnosis of hypertension. This study identified four themes demonstrating the impact of the refugee ban on Syrian and Iraqi refugees resettled in San Diego, CA. The refugee ban and its associated consequences negatively impacted the resilience capacity of refugee participants to manage their hypertension health challenge through family, community, individual, and physiological factors in line with the society to cells framework. The refugee ban negatively impacted refugees’ resilience to hypertension physiologically through the worsening of their mental health and chronic disease. It also impacted their resilience to hypertension individually through worsening their self-perception. Their resilience to the challenge of hypertension was also impacted through community factors, like the perpetuation of Islamophobia, xenophobia, and discrimination. Participants overwhelmingly discussed the impact of the refugee ban in the context of family separation. Many participants shared stories of not being able to reunite with loved ones for several years due to refugees’ application suspensions. Participants mentioned a multitude of ways in which this ban impacted both their physical and mental health - sharing that it resulted in feelings of anxiety, depression, more hospital visits, and the exacerbation of their hypertension.

Hypertension is one of the most prevalent chronic conditions, an important risk factor for cardiovascular disease, and is the number one killer of people in the United States and worldwide (19). Low- and middle-income countries have witnessed significant increases in the prevalence of hypertension and poorer blood pressure control. Refugees, despite being at an all-time high number globally, are rarely included in cardiovascular research (35). Our study is among the first to include refugees in hypertension research where we found links between discriminatory policies and hypertension risk and control. One review article found racial discrimination to be a risk factor for hypertension (10). Awad et al. developed a model of cumulative trauma for MENA individuals divided into macrolevel and microlevel domains of trauma, summarizing how MENA Americans’ feelings of discrimination or marginalization have clear adverse effects on mental and physical health. Applying this model to develop interventions to address factors at both the micro-level (e.g., clinical and counseling efforts to help process historical traumas) and macro-level (e.g., advocacy and policy efforts) can improve psychological well-being among this population (36).

While this study investigated the impact of the refugee ban on hypertensive participants and searched for the linkages between hypertension and the refugee ban, the majority of the emerging themes discussed the impact of the ban with respect to worse mental health and increased stress. It is important to note, however, that the implications of poor mental health on hypertension are plenty. One review article investigating the links between blood pressure and mental illness found mental illnesses to be associated with increased blood pressure variability in young- and middle-aged adults (37). With regards to stress, it has been documented in the literature that stress increases the severity and prevalence of hypertension (38). The implications of stress on hypertension are quite serious. Physiologically, stress can result in blood pressure spikes which may in turn result in damage to the blood vessels and, subsequently, heart attacks and strokes (39).

Though the ban has been lifted, the sequelae are still being felt by many refugee communities who have not yet been reunited with their families. Family reunification is possibly the single most important remedy to the damage caused by the refugee ban. One study in Germany found that family separation among refugees contributes to worse mental health conditions (40). A longitudinal cohort study in Denmark found that refugee men awaiting reunification with their wives and children were at an increased risk of mental disorders (41). Additionally, not only are adverse psychosocial experiences involved in the onset of hypertension, parental separation has been associated with the onset of hypertension in women separated from their parents (42, 43). Rebuilding the United States Refugee Admissions Program is a tangible first step in coordinating family reunification to return United States refugee resettlement rates to their pre-2017 levels. Additionally, due to growing global geopolitical instability and the consequences of climate change, we can expect an exponentially increasing refugee population. Necessary measures must be taken at local, national, and international levels to coordinate a unified response and ensure appropriate capacity to address the needs of this vulnerable population (44).

It is also important to consider the types of support for families once they have been reunified. It is important to organize strategies for reunification that are trauma-informed and can address the mental health concerns faced by separated families up until reunification. A narrative review paper found that community-based trauma-informed care programs following immigrant family reunification are successful in guiding families to reconnect after a prolonged separation (45, 46). Some elements that aided in the success of these programs included collaboration between healthcare providers and education specialists, creating a support group environment, incorporating trauma-informed principles, and partnership with community establishments and liaisons. These factors were identified as important for eliminating the health disparities faced by this population and for building a family reunification program that addresses the mental health concerns of this population (46).

Lack of integration into the host community was cited as an important challenge for our study population, despite almost a decade since resettling in San Diego. Refugee integration must consider this population’s cultural and religious norms and respect their desire to preserve their heritage and culture. One study examining integration and mental health in Arab refugee populations resettled in a Western country found that xenophobia can result in social disconnection between refugees and host groups, and negatively impact integration (47). Furthermore, family reunification greatly affects refugee ability to integrate into the community. This paper also found that worry about family members left behind in their home countries can considerably limit key aspects necessary for the integration of refugees like learning English or having the mental capacity to go about daily life.

Many participants reported low self-perception as a result of the refugee ban and their new identity as resettled refugees in the United States. Older refugees have an increasingly difficult time integrating into the host community. This is due to factors like self-pride and self-efficacy being hinged on their refugee experience. It is necessary to find ways to increase personal agency and independence in this group, which can aid in increasing the sense of self-efficacy. Rather than employment, increasing the involvement of older refugees in community-based activities would be fruitful. For example, one study examined the impact of community gardens on refugees’ physical and mental health and found that refugees reported better physical and emotional benefits from gardening, as well as an improved sense of identity. Refugees also found this intervention helpful for coping with past traumas (48).

The physiological response to discrimination is well documented. Stressful experiences, such as experiencing discrimination, stimulate the release of cortisol and catecholamines through the activation of the autonomic nervous system. Consistent activation of the autonomic nervous system may contribute to chronically elevated blood pressure, thus predisposing to long-term morbidity and mortality (49). Racism has been linked in the literature to poorer hypertension management behaviors such as poorer medication adherence. Prior literature has linked reported racial discrimination in other minority populations such as African Americans to poorer medication adherence which was mediated by trust in physicians (50). Although trust in physicians has not emerged as a theme in our sample, there is literature documenting prevalent poorer physician-patient relationships and lack of trust in the case of refugees (51). Further investigation is warranted to understand the mediators of these behaviors or the psychological underpinnings behind them.

To our knowledge, this is the first study to examine the impact of the refugee ban on Syrian and Iraqi refugees with diagnosed hypertension resettled in San Diego. Additionally, this study had a large sample size for a qualitative study, which strengthens the validity of our research. However, our study has some limitations. We only collected religious affiliations of participants during a sub-study investigating the impact of fasting during Ramadan on hypertension and, therefore, do not have data for all participants’ religious affiliations. It would be worthwhile for future studies to explore the experiences of discrimination noted in this study in a more systematic way to build this literature base. Additionally, due to the sensitive nature of the topics discussed in the interviews, our results may be subjected to social desirability bias which may have resulted in participants withholding the extent to which the refugee ban has impacted their health. However, the interviewers were trained to ensure the comfort of the participants and emphasize the confidential nature of the interview. Also, while we partnered with FHCSD because they are one of the largest healthcare providers serving refugee groups in San Diego, it is still possible that we were not able to reach other potential participants that are not linked to healthcare. And, it is worth noting that the high decline rate for participation during our recruitment could have prevented access to more variable responses in our data. Lastly, considering this study only looked at refugees from Iraq and Syria, we are not able to generalize the experiences of the refugees from the 7 countries on the ban list. Future studies should assess the experiences of refugee groups from other countries impacted by the 2017 refugee ban in other states to ensure generalizability.

Considering the extent to which this field of literature is understudied, it is worthwhile for future investigations in refugee health to extend their scope and build on the findings of this study. It is worth noting in this study that participants did not discuss the impact -if any- of the refugee ban on their lifestyle behaviors and habits. The potential implications of lifestyle behaviors on hypertension management and resilience may be significant and is worthwhile for future studies to investigate these associations. Additionally, considering refugee groups are burdened with many chronic physical health conditions, future studies investigating the impact of the refugee ban on the health of refugees should also study its impact on other chronic diseases like diabetes. Refugees have significantly increased risk of developing diabetes compared to native-born populations (52). Experiences of stressful events like the refugee ban can exacerbate chronic diseases like diabetes (53). Investigations into these potential associations are warranted.

## Data availability statement

The raw data supporting the conclusions of this article will be made available by the authors, without undue reservation.

## Ethics statement

The studies involving human participants were reviewed and approved by Institutional Review Board at the University of California, San Diego. The patients/participants provided their written informed consent to participate in this study.

## Author contributions

TA-R and BA conceived the study. BA, LB, DA-B, and TA-R collected data. BA, LB, and RA analyzed the data. BA and LB drafted the manuscript. All authors contributed to the article and approved the submitted version.

## Funding

This project was made possible by funding from the National Institutes of Health (National Heart, Lung, and Blood Institute grant # K23HL148530).

## Conflict of interest

The authors declare that the research was conducted in the absence of any commercial or financial relationships that could be construed as a potential conflict of interest.

## Publisher’s note

All claims expressed in this article are solely those of the authors and do not necessarily represent those of their affiliated organizations, or those of the publisher, the editors and the reviewers. Any product that may be evaluated in this article, or claim that may be made by its manufacturer, is not guaranteed or endorsed by the publisher.

## Appendix A

1. Date of birth
2. Gender?
  - Male
  - Female
  - Other
3. Country of origin?
  - Syria
  - Iraq
4. When did you resettle to the United States?
5. Are you employed?
  - Yes
  - No
6. Annual household income?
  - Less than $15,000
  - $15,001–$25,000
  - $25,001–$35,000
  - $35,001–$50,000
  - $50,000 or higher
7. What is the highest level of education you have attained?
  - Less than high school
  - High school
  - Vocational certificate
  - Bachelor’s degree
  - Post-graduate degree or higher
8. Are you able to read and write in Arabic?
  - Yes
  - No
9. Are you able to read and write in English?
  - Yes
  - No
10. If you think about your community how many close friends do you have?
  - 1
  - 2
  - 3
  - 4
  - 5 or more
11. In what ways has the refugee experiences impacted your hypertension management and your overall health including social relationships? Probe: Has the refugee ban or policies impacted your health or healthcare access?
